# The Healing of Arteries

**Published:** 1887-01

**Authors:** 


THE HEALING OF ARTERIES AFTER LIGATURE IN MAN AND ANIMALS, by J. Collins Warren, M. D., Assistant Professor of Surgery, Harvard University, Surgeon to the Massachusets General Hospital; member American Surgical Association; Honorary Fellow Philadelphia Academy of Surgery. One Volume. 184 pages. Superbly illustrated with 12 full page plates in black and colors. Parchment muslin binding. Price, $3.25. William Wood & Co., New York.

This book fills a niche in medical literature. The author says: "The study of this important subject has been carried on at various times in a fragmentary way, and this work undertakes to treat of it in a more comprehensive and complete manner. The investigations were made in Harvard Medical School, where great facilities were afforded, especially for studying the different phases through which each tissue passes from the moment of ligature, until a complete healing has taken place.

Neatly bound and printed in clear type, the little book will be found very attractive.

